# IL-1RT1 signaling antagonizes IL-11 induced STAT3 dependent cardiac and antral stomach tumor development through myeloid cell enrichment

**DOI:** 10.18632/oncotarget.2707

**Published:** 2015-01-06

**Authors:** Jon N. Buzzelli, Dan I. Pavlic, Heather V. Chalinor, Louise O'Connor, Trevelyan R. Menheniott, Andrew S. Giraud, Louise M. Judd

**Affiliations:** ^1^ Murdoch Children's Research Institute, Royal Children's Hospital, Parkville, Victoria, 3052 Australia; ^2^ Department of Paediatrics, University of Melbourne, Royal Children's Hospital, Parkville, Victoria, 3052 Australia

**Keywords:** Mucosal immunity, cancer immunobiology, cytokines, IL-11, STAT3, IL-1, MDSCs

## Abstract

IL-1 is key driver of gastric tumorigenesis and is a downstream target of IL-11 signaling. Recently, IL-1 cytokines, particularly IL-1β, have been flagged as therapeutic targets for gastric cancer treatment. Here, we assess the requirement for IL-1 signaling in gastric tumorigenesis. gp130^757FF^ xIL-1RT1^−/−^ mice were generated to determine the pathological consequence of ablated IL-1 signaling in the IL-11 dependent gp130^757FF^ mouse model of gastric tumorigenesis. Gastric lesions in gp130^757FF^ xIL-1RT1^−/−^ mice were increased in incidence and size compared to gp130^757FF^ mice. Proximal gastric lesions originated from the cardiac region and were associated with elevated STAT3 activation, loss of specialized gastric cells and a modulated immune response including increased expression of TNF-α and MDSC associated genes. Administration of IL-11 to IL-1RT1^−/−^ mice showed similar changes to gp130^757FF^ xIL-1RT1^−/−^ mice. Spleens from IL-11 treated wildtype mice showed an enrichment of MDSC and gp130^757FF^ xIL-1RT1^−/−^ mice had increased MDSCs in the stomach compared to gp130^757FF^ mice. Furthermore, crossing TNF-α^−/−^ to gp130^757FF^ mice resulted in reduced lesion size. We conclude that IL-1 signaling antagonizes IL-11/STAT3 mediated pathology and the genetic deletion of IL-1RT1 results in increased tumor burden. We provide evidence that a likely mechanism is due to IL-11/STAT3 dependent enrichment of MDSCs.

## INTRODUCTION

Historically gastric cancers are categorized into 2 main types according to phenotypic and histological characteristics; diffuse carcinomas and intestinal-type gastric cancers (IGC) [1]. IGC develop incrementally after the initial *Helicobacter pylori* (*H. pylori*) infection and acute inflammation, progressing through chronic inflammation, and metaplasia to neoplasia [2]. IGC occur with persistent *H. pylori* infection, most prominently in the distal gastric antrum and also in the cardia. The cardia is a discrete region of glands distinct from the fundus or body of the stomach, located at the gastro-esophageal junction (GEJ) in humans [3], or the limiting ridge between the squamous esophagus and forestomach in mice [4]. These glands are similar to antral glands in structure, being composed primarily of mucous cells, with other specialized cell types being absent. The normal function of cardiac glands is not well defined, nonetheless recent large scale retrospective population studies in humans have shown that cancers of the gastroesophageal junction are increasing in prevalence [5], and that patients with GEJ cancers have reduced life expectancy compared to those with antral IGC [6].

Numerous cytokines have enhanced expression during chronic *H. pylori* infection and subsequent gastric disease progression, in particular members of the IL-1 and IL-6 families. Elevated levels of these cytokines in the absence of other mitigating factors, including *H. pylori*, cause gastric pathology in murine models [4, 7, 8]. Clinical studies have demonstrated a strong correlation between IL-1β polymorphisms and predisposition to gastric cancer development [8–10]. In mice, IL-1β is increased following *H. pylori* infection [10], and transgenic over-expression of IL-1β using the H/K ATPase promoter caused gastric inflammation and dysplasia [8]. More recently, the Epstein-Barr virus promoter/IL-1β transgenic mouse was shown to develop cardiac and esophageal pathology [4]. The link between IL-1β expression and gastric cancer progression in humans and murine models, has resulted in IL-1 receptor blockade being suggested as a novel therapeutic target in the effort to combat gastric cancer progression [11, 12].

IL-1β belongs to a large family of cytokines of which the best characterized are IL-1α, IL-1β, IL-18 and IL-33 [13]. IL-1α and IL-1β bind to their shared receptor, the type 1 IL-1 receptor (IL-1RT1), which subsequently dimerizes with the IL-1R receptor accessory protein (IL-1RAP) resulting in activation of NF-ĸB [14]. The functions of IL-1α and IL-1β are similar in many aspects [13], however IL-1α has the capacity to distinguish between necrosis and atrophy and can be released from damaged cells to promote an immune response [15]. Less is known about the involvement of IL-1α in gastric cancer progression, however IL-1α expression is increased in *H. pylori* associated pathology [16] and elevated IL-1α in gastric tumors has been associated with liver metastasis [17].

The IL-6 family member IL-11 is a pleiotrophic cytokine [7], and has elevated expression in association with gastric cancer development [18, 19]. IL-11 signals via the IL-11Rα/gp130 receptor complex to activate genes involved in proliferation, angiogenesis, inflammation and inhibition of apoptosis [20, 21]. gp130^757FF^ mice contain a knock-in mutation at Y757 of gp130, where a tyrosine has been substituted with phenylalanine, preventing both the phosphatase SHP2 and the negative regulator of STAT3, SOCS3 from binding and consequently resulting in chronic hyper-activation of STAT3 [21]. gp130^757FF^ mice spontaneously develop distal (antral) stomach tumors, which phenocopy chronic *H. pylori*-induced pathology, particularly the key initiating event, pan-gastritis including an abundance of myeloid derived cells [20, 21]. Furthermore, gp130^757FF^ pathology is absolutely dependent on IL-11 signaling [19]. IL-11 expression is increased in multiple mouse models of progressive gastric pathology [19], and in human gastric tumors [19, 22]. Additionally, IL-11 causes profound atrophy in the stomach [7], with a prominent downstream target being IL-1β [8].

The aim of our study was to dissect the individual contributions of oncogenic signaling mediated by gp130 and IL-1RT1 ligands in IL-11/STAT3 mediated gastric cancer development. In addition we evaluated the suitability of IL-1 as novel therapeutic target for gastric cancer. Here we show that IL-1RT1-mediated signaling is dispensable for antral tumor development in the gp130^757FF^ mouse, as well as IL-11-induced fundic atrophy and metaplasia. In fact, in the absence of IL-1RT1 signaling gastric pathology develops more rapidly and extensively in both the antrum and gastric cardia suggesting that IL-1RT1 signaling may minimize tumor burden by antagonizing IL-11/STAT3 mediated pathology.

## RESULTS

### Myeloid-derived immunocytes up-regulate the IL-1 signaling network in the antrum of gp130^757FF^ mice

**Figure 1:**
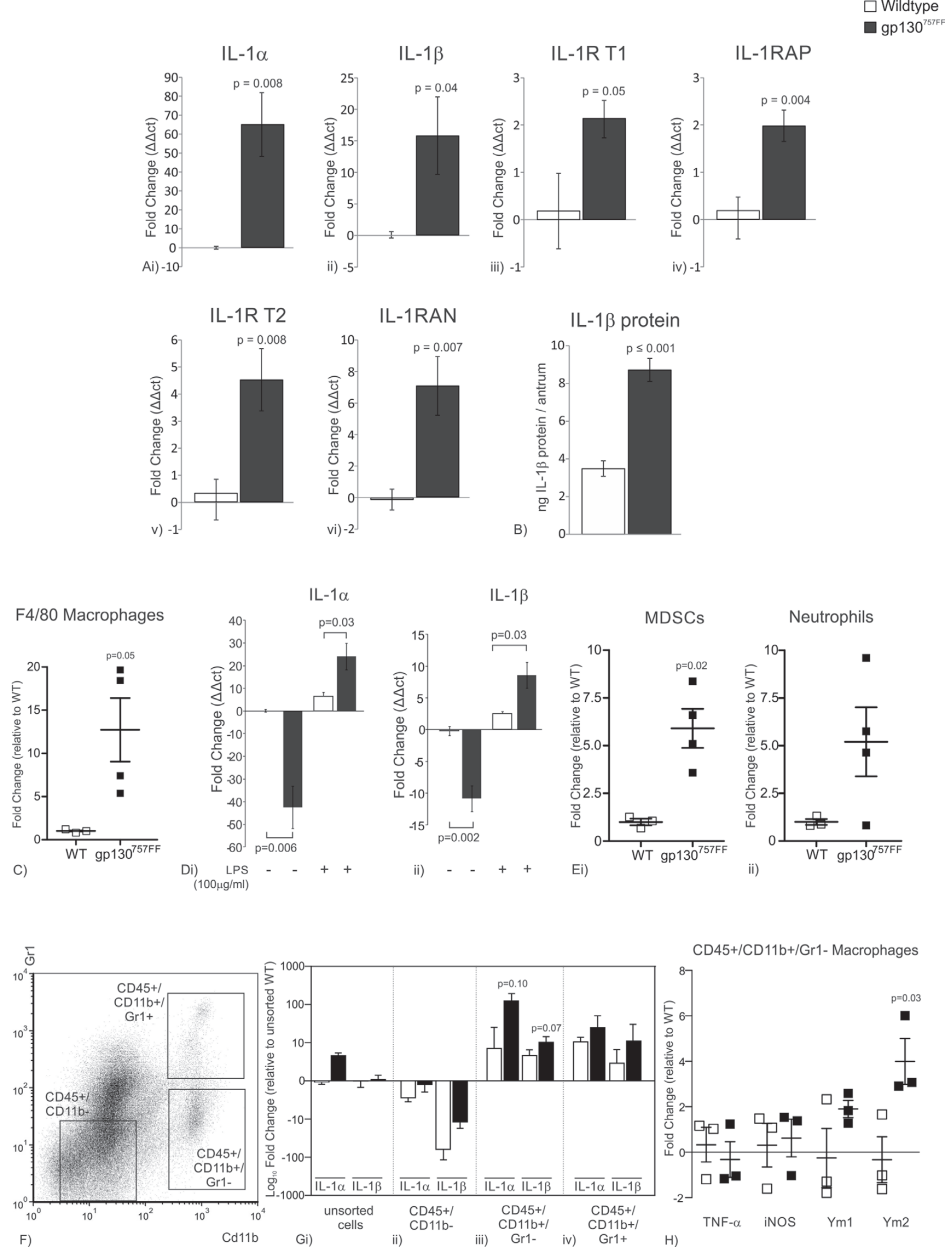
mRNA expression of IL-1RT1 ligands and associated proteins in the distal stomach of 30 week old gp130^757FF^ compared to wildtype mice **(A)**(i) IL-1α, (ii) IL-1β, (iii) IL-1R type 1, (iv) IL-1RAP, (v) IL-1R type 2 and (vi) IL-1RAN. **(B)** Protein expression of (i) IL-1β in the distal stomach of 30 week old gp130^757FF^ compared to wildtype mice. **(C)** Proportion of F4/80 macrophages in the stomach of 12 week gp130^757FF^ mice compared to wildtype mice. **(D)** mRNA expression of (i) IL-1α and (ii) IL-1βin peritoneal macrophages isolated from either wildtype or gp130^757FF^ cultured in the presence or absence of LPS (100ng/μl). **(E)** Proportion of (i) MDSC and (ii) neutrophils in the stomach of 12 week gp130^757FF^ mice compared to wildtype mice. **(F)** Presentation of how CD45^+^ gastric immunocytes were sorted for CD11b and Gr1. **(G)** mRNA expression of IL-1α and IL-1β of cell sorted populations; (i) unsorted cells, (ii) CD45^+^ /CD11b^−^, (iii) CD45^+^ /CD11b^+^ /Gr1^−^ and (iv) CD45^+^ /CD11b^+^ /Gr1^+^. **(H)** mRNA expression of M1 macrophages markers (TNF-α and iNOS) and M2 macrophage markers (Ym1 and Ym2). Bars are means ± SEM, *p*-values are presented for statistically significant changes (*p* < 0.05).

gp130^757FF^ mice spontaneously develop antral stomach tumors caused by constitutive activation of STAT3 and by 30 weeks of age the antrum of a gp130^757FF^ mouse is largely tumor tissue. IL-11 is essential for gp130^757FF^ induced tumorigenesis [19, 20], however the importance of another cytokine, IL-1β, and its signaling pathway, has not been quantitatively assessed in this model. IL-1α and IL-1β expression were increased in 30 week old gp130^757FF^ mice compared to wildtype littermates, (65.1 ± 16.8 and 15.8 ± 6.1 fold respectively; Fig. 1Ai&ii). The expression of IL-1RT1 and its accessory protein (IL-1RAP) were also increased (2.1 ± 0.4 and 2.0 ± 0.3 fold respectively; Fig. 1Aiii&iv) as were the IL-1R decoy receptor (IL-1RT2) and the IL-1R antagonist protein (IL-1RAN)(4.17 ± 1.1 and 6.31 ± 1.7 fold respectively; Fig. 1Av&vi). IL-1β protein was also analyzed by ELISA assay and was increased in the antrum of gp130^757FF^ mice compared to WT mice (WT: 3.5 ± 0.4, gp130^7575FF^ : 8.7 ± 0.6; Fig. 1B). Together these data suggest that IL-1 signaling may contribute to antral tumor development in gp130^757FF^ mice.

Activated macrophages have the capacity to secrete pro-inflammatory IL-1 cytokines, and since inflammation is crucial for gp130^757FF^ tumor development [20] we assessed whether activated macrophages contribute to elevated IL-1α and IL-1β expression in 30 week old gp130^757FF^ mice. Relative to wildtype mice, gp130^757FF^ stomachs had a large increase in F4/80 positive macrophage infiltrate (12 ± 3.7 fold; Fig. 1C). Peritoneal macrophages extracted from gp130^757FF^ mice had strongly attenuated IL-1α and IL-1βmRNA expression compared to wildtype macrophages (−42.4 ± 9.4 and −10.8 ± 2.0 fold respectively; Fig. 1Di&ii). In wildtype macrophages LPS stimulation increased IL-1α and IL-1βmRNA expression by 6.5 ± 1.8 and 2.4 ± 0.3 fold respectively, whilst in stimulated gp130^757FF^ macrophages IL-1α and IL-1β were further increased by 24.1 ± 5.9 and 8.5 ± 2.0 fold respectively (Fig. 1Di&ii). These data suggest that IL-1RT1 ligands contribute to the development of gp130^757FF^ gastric tumors and that activated macrophages are a potential source of their expression.

While macrophages likely contribute a significant proportion of the gastric mucosal IL-1 ligand pool, other cellular components, including MDSCs, have been demonstrated to contribute to gastric cancer development and IL-1 cytokine production [8]. We therefore quantified MDSC infiltrate as well as another myeloid-derived immunocyte population, neutrophils, in gp130^757FF^ stomachs compared to wildtype mice (Fig. 1E). Relative to wildtype mice, gp130^757FF^ stomachs had an increase in CD11b^+^ /Gr-1^INT^ MDSC infiltrate (5.9 ± 1.0 fold; Fig. 1Ei) and CD11b^+^ /Gr-1^HIGH^ neutrophils (5.2 ± 1.8 fold; Fig. 1Eii); however these increases were not as substantial as infiltrating macrophages (Fig. 1C).

To extend our understanding of which immunocyte populations contribute to IL-1 cytokine enrichment in gastric lesions of gp130^757FF^ mice, gastric immunocytes from wildtype (*n* = 3) and gp130^757FF^ mice (*n* = 3) were sorted. Cell populations collected were as follows (Fig. 1F&1G): CD45^+^/CD11b^−^ cells which are inclusive of CD4^+^ T cells, CD8^+^ T cells, B cells and dendritic cells; CD45^+^/CD11b^+^/Gr-1^−^ cells (macrophages); and CD45^+^/CD11b^+^/Gr-1^+^ cells (Neutrophils and MDSCs). The mRNA expression of IL-1α and IL-1β was measured in these cells populations by QRT-PCR and compared to wildtype unsorted cells. Unsorted gp130^757FF^ cells had an increase in IL-1α and IL-1β mRNA expression compared to wildtype controls consistent with findings from whole stomachs (IL-1α: 4.7 ± 0.8; IL-1β: 0.5 ± 0.9; Fig. 1Gi). Relative to unsorted wildtype cells, wildtype CD45^+^/CD11b^−^ cells had reduced IL-1α and IL-1β mRNA expression by –3.0 ± 0.6 and –84.3 ± 36.0 respectively (Fig. 1Gii). This decrease was consistent in gp130^757FF^ CD45^+^/CD11b^−^ cells. In gp130^757FF^ CD45^+^/CD11b^−^ cells IL-1α and IL-1β were decreased by –2.0 ± 0.4 and –11.5 ± 1.6 respectively (Fig. 1Gii). In contrast to isolated CD45^+^/CD11b^−^ cells, CD45^+^/CD11b^+^/Gr-1^−^ cells and CD45^+^/CD11b^+^/Gr-1^+^ cells had enhanced IL-1α and IL-1β expression (Fig. 1Giii&iv). Relative to wildtype unsorted cells, wildtype CD45^+^/CD11b^+^/Gr-1^−^ cells had an increase in IL-1α and IL-1β expression by 17.6 ± 11.7 and 5.1 ± 1.5 fold respectively (Fig. 1Giii). This increase was even greater in gp130^757FF^ CD45^+^/CD11b^+^/Gr-1^−^ cells (IL-1α: 147.3 ± 55.0 fold; IL-1β: 11.6 ± 3.9 fold; Fig. 1Giii). Similarly, wildtype CD45^+^/CD11b^+^/Gr-1^+^ cells had an increase in IL-1α and IL-1β expression by 11.3 ± 2.6 and 4.5 ± 3.8 compared to unsorted wildtype cells (Fig. 1Giv). Once again this increase was more pronounced in gp130^757FF^ CD45^+^/CD11b^+^/Gr-1^+^ cells (IL-1α: 41.1 ± 27.7 fold and IL-1β: 25.8 ± 19.6 fold; Fig. 1Giv). Considering the large macrophage infiltrate in gp130^757FF^ stomachs we wanted to determine how these macrophages were polarized, therefore CD45^+^/CD11b^+^/Gr-1^−^ cells (macrophages) were assessed for M1 and M2 markers by QRT-PCR. Relative to wildtype sorted macrophages, gp130^757FF^ macrophages had no change in the M1 genes TNF-α and iNOS [23] (Fig. 1H). In contrast the M2 genes, Ym1 and Ym2 [23], were both increased in gp130^757FF^ macrophages com-pared to wildtype macrophages by 1.9 ± 0.4 and 4.0 ± 1.0 respectively (Fig. 1H). Collectively, these data demonstrate that macrophages are highly abundant in gp130^757FF^ lesions, express high levels of IL-1α and IL-1β and therefore are the greatest contributors of IL-1α and IL-1β enrichment in gp130^757FF^ gastric lesions. Additionally, MDSCs and neutrophils are also present within gp130^757FF^ gastric lesions and have increased IL-1α and IL-1β mRNA expression, however not to the extent of macrophages.

We also isolated E-cadherin positive epithelial cells from wildtype and gp130^757FF^ mice and assessed IL-1α and IL-1β mRNA expression however we could not detect a signal for either cytokine (data not shown) suggesting the epithelium does not significantly contribute to increased IL-1α and IL-1β in gp130^757FF^ gastric lesions.

### The loss of IL-1RT1 increases gp130^757FF^ gastric tumor burden

**Figure 2:**
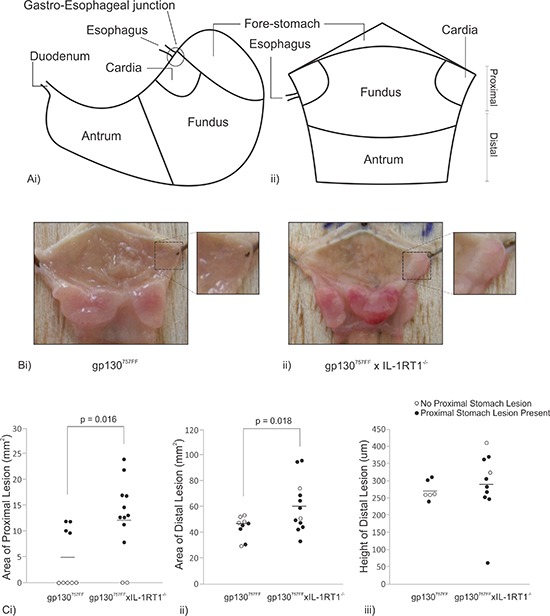
Macroscopic analysis of pathology in 12 week old gp130^757FF^ xIL-1RT1^−/−^ mice compared to gp130^757FF^ mice **(A)** A graphical representation of an intact mouse stomach (i) which has been dissected along the lesser curvature and pinned onto balsa (ii). **(B)** Macroscopic images of a gp130^757FF^ mouse stomach (i) and a gp130^757FF^ xIL-1RT1^−/−^ mouse stomach (ii). **(C)** Macroscopic analysis of lesion area in the proximal stomach (i) and distal (ii) and microscopic analysis of distal lesion height (iii). *p*-values are presented for statistically significant changes (*p* < 0.05).

In order to assess the contribution of IL-1 signaling to tumor burden directly, gp130^757FF^ mice were crossed with IL-1RT1^−/−^ (receptor utilized by both IL-1α and IL-1β) mice. Stomachs were dissected and opened along the lesser curvature (Fig. 2A). As expected gp130^757FF^ mice develop tumor lesions in the antral stomach averaging 44.3 ± 2.3mm^2^. Surprisingly, gp130^757FF^ xIL-1RT1^−/−^ mice develop considerably larger antral lesions cumulatively measuring 60.6 ± 5.6mm^2^ (Fig. 2B&2Cii). This data demonstrates that IL-1 signaling is not required for gp130^757FF^ tumorigenesis and that an absence of IL-1 signaling enhances antral tumor growth.

gp130^757FF^ xIL-1RT1^−/−^ mice also developed lesions in the proximal stomach which originate from the cardiac glands near the gastro-esophageal junction and will herein be referred to as cardiac lesions (compare Fig. 2Bi and 2Bii). Cardiac lesions were present in 83.3% of gp130^757FF^ xIL-1RT1^−/−^ mice [*n* = 12] compared to 44.4% of gp130^757FF^ mice [*n* = 9]; Fig. 2Cii). When cardiac lesions were present in gp130^757FF^ mice they were considerably smaller than those in gp130^757FF^ xIL-1RT1^−/−^ mice (5.0 ± 2.0mm^2^ and 12.7 ± 2.1mm^2^ respectively; Fig. 2Cii). Interestingly, cardiac lesions did not influence distal tumor height (Fig. 2Ciii), suggesting that proximal and distal lesions develop independently.

**Figure 3:**
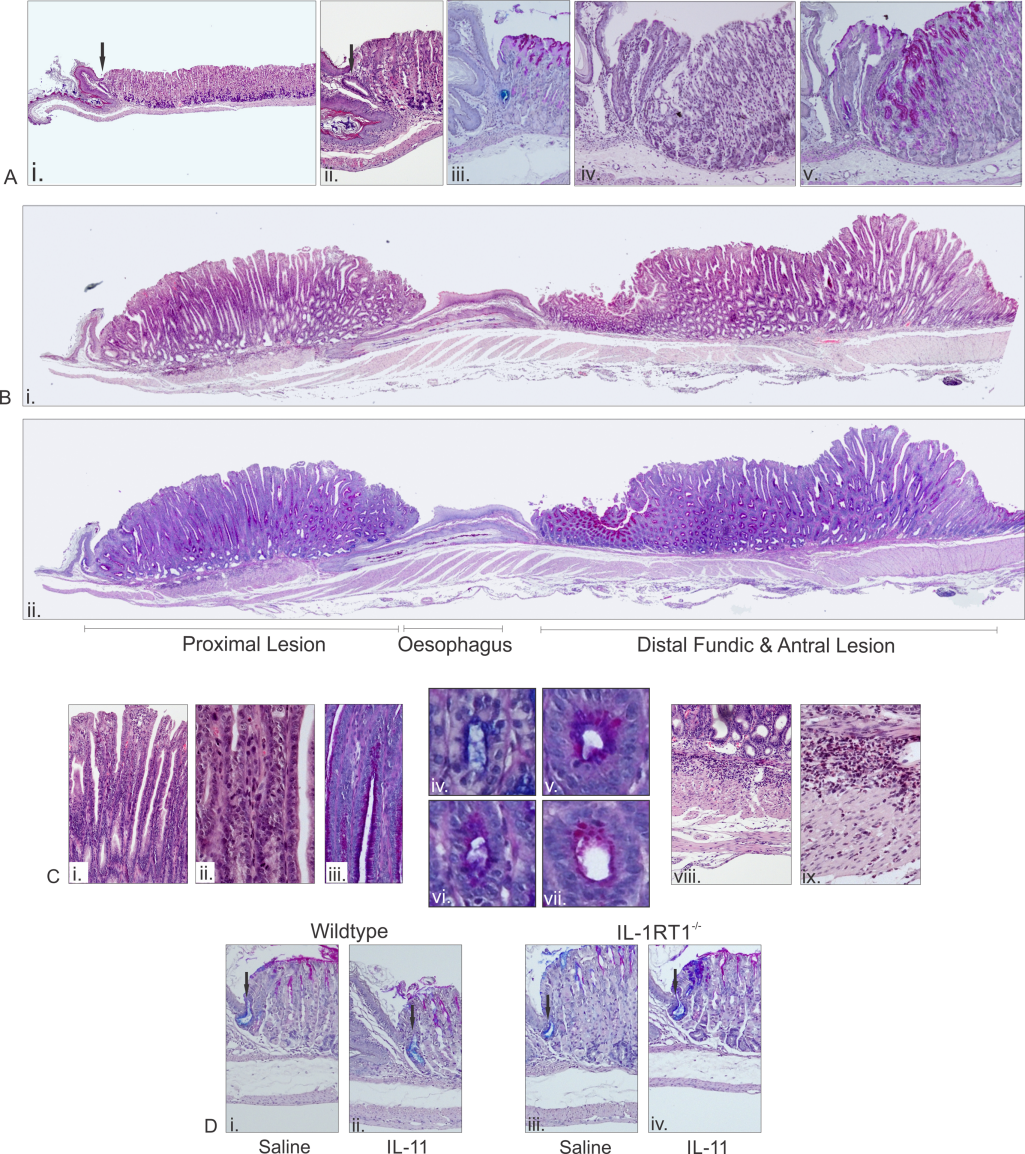
Microscopic analysis of pathology in 12 week old gp130^757FF^ xIL-1RT1^−/−^ mice and in WT and IL-1RT1^−/−^ mice administered with IL-11 for 7 days **(A)** H&E landscape of a wildtype proximal stomach (Fundus/Cardia) (i), normal cardia H&E (ii), and AB-PAS (iii). A developing lesion in the gp130^757FF^ xIL-1RT1^−/−^ cardia regions H&E (iv) and AB-PAS (v). **(B)** A pronounced lesion in the gp130^757FF^ xIL-1RT1^−/−^ stomach H&E (i) and AB-PAS (ii). **(C)** Glandular structure gp130^757FF^ xIL-1RT1^−/−^ stomach (H&E; i&ii, AB-PAS; iii–vi) and associated submucosal infiltrate (H&E; viii&ix). **(D)** Characteristic cardia in WT mice treated with saline (i) or IL-11 (ii) and in IL-1RT1–/– mice treated with saline (iii) or IL-11 (iv). Arrows in images indicate cardiac glands.

Histological assessment of gp130^757FF^ xIL-1RT1^−/−^ stomachs supports a cardiac gland origin for the proximal stomach lesions. In wildtype mice the cardia is typically restricted to a single gland directly adjacent to the fore-stomach (arrow in Fig. 3Ai&ii). The cells within this gland are cuboidal epithelial cells and resemble the mucous cells of antral glands, reinforced by the presence of acidic carbohydrates evident with Alcian blue staining (Fig. 3A iii). Histologically, parietal, chief and mucous neck cells are absent from the cardiac glands. Examination of the proximal stomach from a gp130^757FF^ xIL1RT1^−/−^ mouse demonstrates that the most significantly affected glands are the cardiac glands adjacent to the forestomach. These cardiac glands expand and become hyperplastic with associated inflammatory cells. Directly adjacent to the abnormal cardia are normal fundic glands (Fig. 3Aiv&v). With larger cardiac lesions the origin of the lesion is no longer as apparent since the entire proximal stomach is affected and proximal lesions become continuous with distal lesions (Fig. 3Bi&ii). Characteristics of the proximal lesions include near complete atrophy, foveolar hyperplasia and some dysplasia (Fig. 3Ci–iii), accompanied by a mix of mucous cells (Fig. 3Civ–vii) and moderate inflammation (Fig. 3Cviii&ix).

Previously, in a study assessing the effects of maximal gp130/STAT3 activation on the gastric mucosa, we reported that systemically administered IL-11 (20μg/day) caused gastric atrophy and mucus metaplasia through STAT3 activation, and coincident with elevated IL-1β expression [7]. Others have shown that overexpression of IL-1β in the mouse stomach results in tumor induction [4, 8]. Here we administered a significantly lower and more physiologically relevant dose of IL-11 (1μg/day) to wildtype and IL-1RT1^−/−^ mice to determine if IL-1RT1 signaling was required for IL-11 induced changes to the gastric mucosa. Histological assessment of the cardiac and fundic glands following administration of low concentrations of IL-11 demonstrated no obvious changes in either wildtype or IL-1RT1^−/−^ mice (Fig. 3D). Normal cardiac glands stained positive for acidic mucins are indicated in each image (Fig. 3Di–iv).

### IL-11/STAT3 mediates the development of gp130^757FF^ xIL-1RT1^−/−^ cardiac lesions

**Figure 4:**
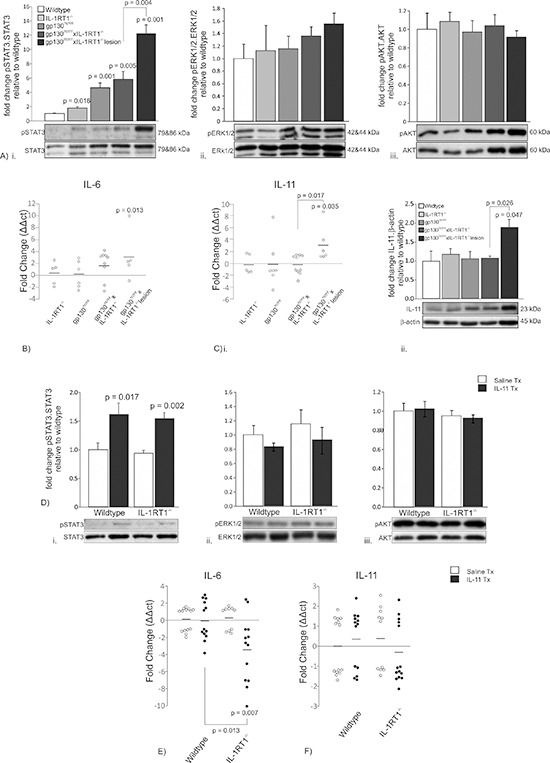
Proximal stomach signaling mechanisms in 12 week old gp130^757FF^ xIL-1RT1^−/−^ mice and, wildtype and IL-1RT1^−/−^ mice administered 1ug of IL-11 daily for 7 days. gp130^757FF^ mice develop gastric pathology because of hyperactivation of STAT3; therefore the expression of gp130 ligands and activations of downstream transcription factors were analyzed **(A)** Immunoblot analysis of STAT3, ERK1/2 and AKT phosphorylation in 12 week old gp130^757FF^ xIL-1RT1^−/−^ mice and expression of **(B)** IL-6 mRNA and **(C)** IL-11 (i) mRNA and protein (ii). **(D)** Immunoblot analysis of STAT3, ERK1/2 and AKT phosphorylation in wildtype and IL-1RT1^−/−^ mice administered 1μg of IL-11 for 7 days and mRNA expression of **(E)** IL-6, and **(F)** IL-11. Bars are means ± SEM, *p*-values are presented for statistically significant changes (*p* < 0.05).

Antral tumor development in gp130^757FF^ mice is caused by IL-11-dependent hyperactivation of STAT3 [19], so we tested whether the cardiac stomach lesions of gp130^757FF^ xIL-1RT1^−/−^ mice also developed as a result of IL-11-induced STAT3 phosphorylation (pSTAT3). pSTAT3/STAT3 in the proximal stomach was elevated in IL-1RT1^−/−^, gp130^757FF^ and gp130^757FF^ xIL-1RT1^−/−^ mice compared to wildtype controls (1.8 ± 0.2, 4.6 ± 0.7 and 5.8 ± 1.1 fold respectively; Fig. 4Ai). Cardiac lesions of gp130^757FF^ xIL-1RT1^−/−^ mice also had a marked increase in STAT3 phosphorylation compared to all other groups suggesting excess STAT3 activation drives cardiac lesion development (12.2 ± 1.3; Fig. 4Ai). Activation of the gp130 complex can additionally induce three alternative transcriptional factors, STAT1, EKR1/2 and AKT, through phosphorylation events [24]. We quantified these signaling molecules in the proximal stomach of gp130^757FF^ xIL-1RT1^−/−^ mice. No activation of STAT1 was observed for any group, whilst the ratio of ERK1/2 and AKT phosphorylation to total protein was not statistically different between groups (Fig. 4Aii&iii), further highlighting STAT3 activation as the driver of cardiac lesion development. To determine the gp130 ligand required for STAT3 activation within the cardiac lesions we measured IL-6 and IL-11 mRNA expression, as well as the IL-11 protein. IL-6 mRNA expression was increased in the cardiac lesions of gp130^757FF^ xIL-1RT1^−/−^ mice (2.9 ± 1.3 fold) however, it was not different to unaffected proximal stomach from gp130^757FF^ xIL-1RT1^−/−^ mice (Fig. 4B). IL-11 mRNA expression was significantly increased 3.2 fold in the gp130^757FF^ xIL-1RT1^−/−^ cardiac lesions compared to wildtype and unaffected gp130^757FF^ xIL-1RT1^−/−^ tissue (Fig. 4Ci). Relative to β-actin, IL-11 protein was also increased in the gp130^757FF^ xIL-1RT1^−/−^ cardiac lesions (1.87 ± 0.22 fold) compared to wildtype and unaffected gp130^757FF^ xIL-1RT1^−/−^ tissue (Fig. 4Cii). We also attempted to determine IL-6 protein levels however were unable to detect IL-6 protein in the fundic or cardiac gastric mucosa of any group using three different IL-6 specific antibodies (sc-1265, Santa Cruz; MP5-20F3, BD Biosciences; MP5-32C11, BD Biosciences). These data suggest that IL-11 but not IL-6 induced STAT3 activation promotes the development of cardiac lesions, a pathological outcome which is more frequent with ablated IL-1 signaling.

### IL-1RT1 signaling does not affect IL-11 signaling in the stomach

To determine the requirement of IL-1 signaling for the pathological effects of IL-11 following its systemic administration, we measured the pSTAT3/STAT3 ratio in the proximal stomach of mice treated with 1μg IL-11/day delivered by mini-osmotic pumps (MOPs). Wildtype (1.62 ± 0.20 fold) and IL-1RT1^−/−^ mice (1.54 ± 0.11 fold) had similar increases in STAT3 phosphorylation following IL-11 administration, suggesting that IL-1 signaling is not required for IL-11 signaling in the stomach (Fig. 4Di). We also tested the activation of STAT1, ERK1/2 and AKT. STAT1 activation could not be detected in any group, and the ratio of ERK1/2 and AKT phosphorylation compared to total protein was not altered (4Dii&iii). As IL-11 administration may promote endogenous expression of gp130 ligands, thereby confounding its effects, we measured the mRNA expression of endogenous gastric IL-6 and IL-11. IL-6 was decreased in IL-11 treated IL-1RT1^−/−^ mice compared to IL-11 treated wildtype mice (–3.5 ± 1.0 fold; Fig. 4E). The expression of IL-11 was not changed in IL-11 treated wildtype or IL-1RT1^−/−^ mice (Fig. 4F). These data show that the loss of IL-1 signaling does not influence IL-11-mediated STAT3 activation. However, consistent with previous reports [4], IL-1RT1 signaling is required for IL-6 expression during STAT3 induced gastric atrophy.

Proximal stomach lesions show transmural inflammation associated with precancerous changes (Fig. 3C) and elevated STAT3 activation with increased IL-11 expression (Fig. 4). We therefore investigated genes involved in immune regulation and epithelial homeostasis. Relative to wildtype, DMBT1 [25] expression was increased in gp130^757FF^ proximal stomach (3.3 ± 1.0 fold), unaffected gp130^FF^ xIL-1RT1^−/−^ proximal stomach (2.8 ± 0.3 fold) and to a much greater extent in gp130^FF^ xIL-1RT1^−/−^ cardiac stomach lesions (34.7 ± 9.0 fold; Supp Fig. 1). Similarly RegIIIβ [26] was increased 5.1 ± 1.7, 19.7 ± 11.3 and 575.0 ± 96.1 fold respectively (Supp Fig. 1). Conversely, GKN1 and GKN2 [27] were decreased in gp130^FF^ xIL-1RT1^−/−^ cardiac stomach lesions 5.6 ± 1.2 and 4.9 ± 0.9 fold respectively (Supp Fig. 1). DMBT1 mRNA expression was increased in IL-11 treated WT and IL-1RT1^−/−^ mice (4.4 ± 1.2 fold 2.8 ± 0.6 fold; Supp Fig. 1), whilst RegIIIβ was not altered (Supp Fig. 1). GKN1 and GKN2 mRNA expression were decreased in IL-11 treated IL-1RT1^−/−^ mice (−1.3 ± 0.2 fold), but not IL-11 treated WT mice (Supp Fig. 1). These data suggests that the development of cardiac stomach lesions is associated with enhanced DMBT1 and RegIIIβ and suppressed GKN1&2 mRNA expression. In addition these effects are potentiated by IL-11 in the absence of IL-1RT1 signaling, underscoring the crucial role played by IL-11 in gastric tumorigenesis.

### gp130^757FF^ xIL-1RT1^−/−^ cardiac lesions show a loss of differentiated gastric cell markers

Since gp130^757FF^ xIL-1RT1^−/−^ cardiac lesions showed extensive atrophic changes we compared the loss of specialized gastric cell types by assessing mRNA expression markers of differentiated fundic cell populations. gp130^757FF^ xIL-1RT1^−/−^ cardiac lesions showed a significant reduction in mRNA expression of markers for parietal cells (H/K ATPase α, H/K ATPase β & CCKBR), chief cells (Mist1), surface mucous cells (Muc5AC) and progenitor cells (BMP4&Lgr5; Supp Fig. 2). The metaplastic SPEM lineage (TFF2) was also reduced in gp130^757FF^ xIL-1RT1^−/−^ cardiac lesions however not significantly (Supp Fig. 2). Administration of low dose IL-11 to wildtype mice did not alter the expression of proximal stomach gastric cell markers, however IL-11 treatment of IL-1RT1^−/−^ mice led to reduced expression of markers of differentiation in a similar pattern to gp130^757FF^ xIL-1RT1^−/−^ mice (Supp Fig. 2). This data supports previous findings which demonstrate IL-11 to induce gastric atrophy [7], a process which appears to be accelerated in the absence of IL-1RT1 signaling as demonstrated by a decrease of mRNA markers of gastric epithelial cells (Supp Fig. 2).

### Systemic administration of IL-11 causes an enrichment of MDSCs

**Figure 5:**
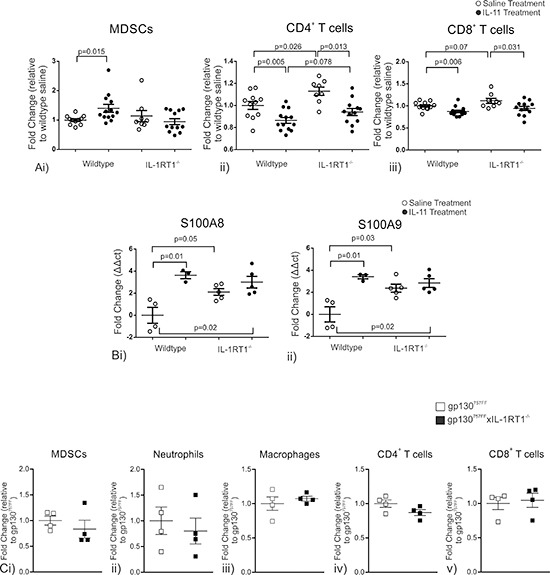
**(A)** Cell populations in wildtype and IL-1RT1^−/−^ mice constitutively administered 1μg IL-11 daily for 7 days; (i) MDSC, (ii) CD4 T cells and (iii) CD8 T cells. **(B)** mRNA expression changes of genes expressed by MDSCs analyzed via QRT-PCR in the spleens of wildtype and IL-1RT1^−/−^ mice constitutively administered 1μg IL-11 daily for 7 days (i) S100A8 & (ii) S100A9. **(C)** Spleen immune cell populations in gp130^757FF^ xIL-1RT1^−/−^ mice compared to gp130^757FF^ mice; (i) MDSCs, (ii) Neutrophils, (iii) Macrophages, (iv) CD4^+^ T cells and (v) CD8^+^ T cells. Bars are means ± SEM, *p*-values are presented for statistically significant changes (*p* < 0.05).

Systemic IL-11 administration caused marked changes to splenic cell populations (Fig. 5A). CD11b^+^/Gr-1^INT^ MDSCs were enriched in wildtype mice treated with IL-11 (WT-control: 1.0 ± 0.04 fold; WT-IL11: 1.4 ± 0.13 fold) but this did not occur in the absence of IL-1 signaling (IL-1RT1-control: 1.03 ± 0.06; IL-1RT1-IL-11:1.05 ± 0.08; Fig. 5Ai). Conversely, IL-11 administration resulted in a reduction in CD4^+^ T cells (WT-control: 1.0 ± 0.04 fold; WT-IL11: 0.86 ± 0.03 fold) and CD8^+^ T cells (WT-control: 1.0 ± 0.03 fold; WT-IL11: 0.86 ± 0.03 fold) in wildtype mice (Fig. 5Aii&iii). Interestingly, basal (no treatment) IL-1RT1^−/−^ mice had an increase in CD4^+^ T cells (WT-control: 1.0 ± 0.04; IL-1RT1-control: 1.14 ± 0.04) and CD8^+^ T cells (WT-control: 1.0 ± 0.03; IL-1RT1-control: 1.13 ± 0.06) compared to wildtype mice, however they showed a similar reduction in CD4^+^ T cells (IL-1RT1-control: 1.14 ± 0.04; IL-1RT1-IL11: 0.095 ± 0.03) and CD8^+^ T cells (IL-1RT1-control: 1.0 ± 0.03; IL-1RT1-IL11: 0.096 ± 0.04) following IL-11 treatment (Fig. 5Aii&iii). Furthermore, the basal mRNA expression of S100A8 and S100A9, established markers of MDSC activity, were increased in the spleen of IL-1RT1^−/−^ mice compared to WT mice (3.6 ± 0.3 and 3.4 ± 0.2 fold respectively; Fig. 5Bi&ii). Following IL-11 administration wildtype mice showed an increase in mRNA expression of S100A8 and S100A9, being 2.1 ± 0.3 and 2.4 ± 0.4 fold respectively (Fig. 5Bi&ii). The basal increase of S100A8 and S100A9 in IL-1RT1^−/−^ mice compared to wildtype controls was not affected by IL-11 administration (Fig. 5Bi&ii). Other MDSC associated genes including MIP2 and Arg-1 were also tested, however they were unchanged between groups (data not shown). In contrast, there were no differences in splenic immunocyte populations between gp130^757FF^ mice and gp130^757FF^ xIL-1RT1^−/−^ mice (Fig. 5C). These data show that systemic IL-11 enhances MDSC enrichment in the spleen whilst reducing CD4^+^ and CD8^+^ T cells. Furthermore, absence of IL-1 signaling confers heightened activity of MDSC cells, evident by an increase in S100A8 and S100A9 mRNA expression, and mimicked by the administration of IL-11 to wildtype mice.

### MDSCs are enriched in the stomach of gp130^757FF^ xIL-1RT1^−/−^ mice compared to gp130^757FF^ mice

**Figure 6:**
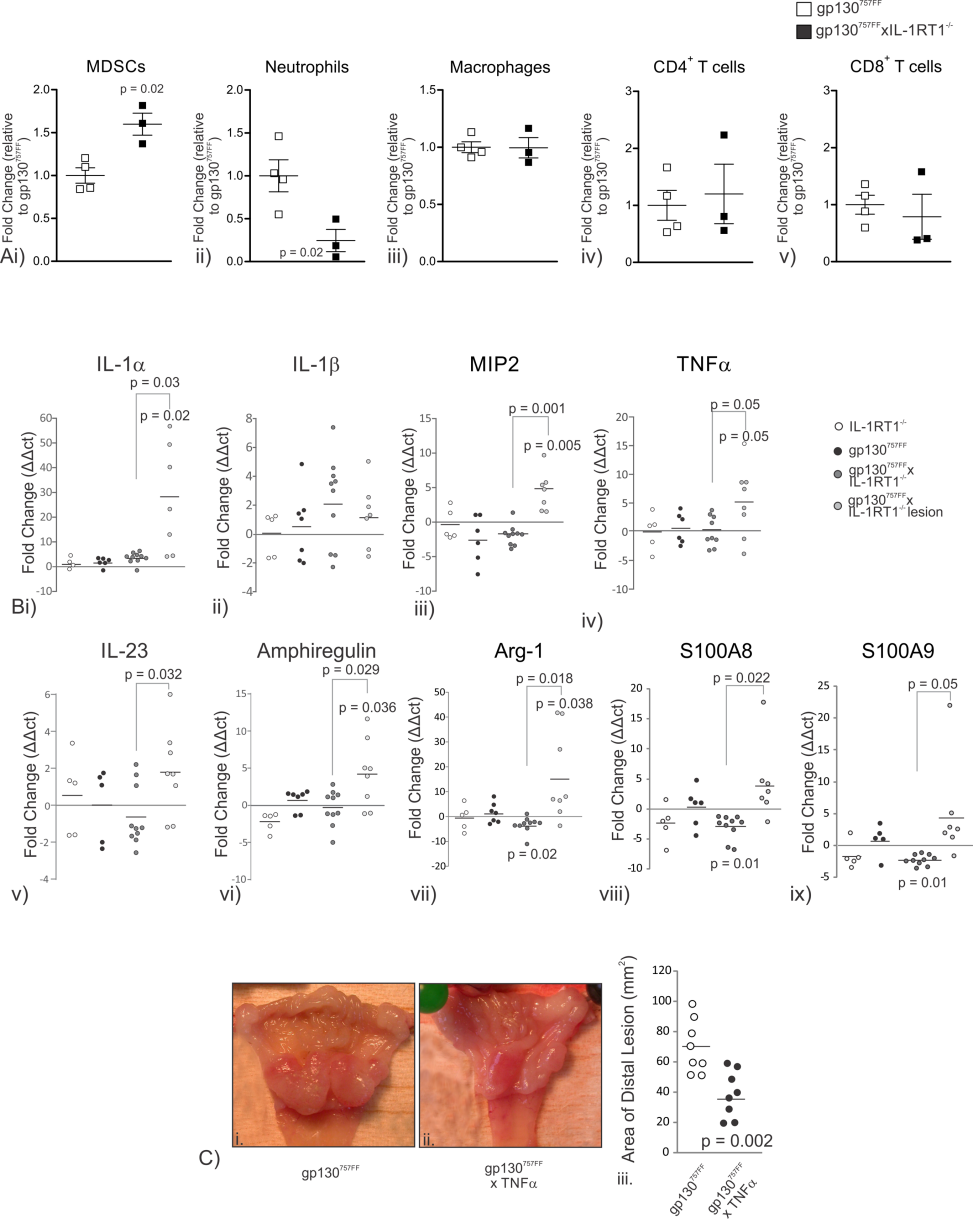
**(A)** Stomach immune infiltrate in gp130^757FF^ xIL-1RT1^−/−^ mice compared to gp130^757FF^ mice; (i) MDSCs, (ii) Neutrophils, (iii) Macrophages, (iv) CD4^+^ T cells and (v) CD8^+^ T cells. **(B)** Proximal stomach mRNA expression of immune modulating genes in gp130^757FF^ xIL-1RT1^−/−^ cohort mice; (i) IL-1α (ii) IL-1β (iii) MIP2 (iv) TNFα (v) IL-23 (vi) Amphiregulin (vii) Arg-1 (viii) S100A8 & (ix) S100A9. **(C)** (i–iii) Assessment of distal tumor size in gp130^757FF^ xTNFα^−/−^ mice compared to gp130^757FF^ mice. *p*-values are as indicated for statistically significant changes (*p* < 0.05).

Gastric tumors are inflammatory in nature therefore we assessed the abundance of the myeloid and T cell populations in the stomachs of gp130^757FF^ xIL-1RT1^−/−^ mice compared to gp130^757FF^ mice (Fig. 6A). Relative to gp130^757FF^ mice, gp130^757FF^ xIL-1RT1^−/−^ mice had an increase in gastric CD11b^+^/Gr-1^INT^ MDSCs (gp130^757FF^: 1.0 ± 0.12; gp130^757FF^ xIL-1RT1^−/−^:1.6 ± 0.13) and a decrease in neutrophils (gp130^757FF^: 1.0 ± 0.22; gp130^757FF^ xIL-1RT1^−/−^: 0.25 ± 0.13; Fig. 6Ai&ii). F4/80 macrophages, CD4^+^ T cells and CD8^+^ T cells were not changed between groups (Fig. 6Aiii–v). These data suggest that increased MDSC infiltrate and deceased neutrophil infiltrate in the stomach of gp130^757FF^ xIL-1RT1^−/−^ mice exacerbates gastric pathology in these mice compared to gp130^757FF^ mice.

gp130^757FF^ mice have increased gastric IL-1α and IL-1β mRNA expression. In gp130^757FF^ xIL-1RT1^−/−^ mice, IL-1α was increased by 3.5 ± 0.7 fold in the unaffected proximal stomach and by 27.2 ± 7.7 fold in cardiac lesions (Fig. 6Bi). In contrast, IL-1β mRNA expression was unchanged (Fig. 6Bii). Considering gastric tumors of gp130^757FF^ mice are associated with increased prevalence of myeloid cells (Fig. 1C&1E), we measured the mRNA expression of MIP2, TNF-α, IL-23 and amphiregulin; four cytokines known to be expressed by myeloid-derived cells. MIP2 and TNF-α mRNA expression were substantially increased in gp130^757FF^ xIL-1RT1^−/−^ cardiac lesions compared to unaffected gp130^757FF^ xIL-1RT1^−/−^ proximal stomach (4.5 ± 1.0 and 5.3 ± 2.2 fold respectively; Fig. 6Biii&iv). IL-23 and amphiregulin expression were also significantly increased in gp130^FF^ xIL-1RT1^−/−^ cardiac lesions compared to unaffected stomach (1.8 ± 0.8 and 4.2 ± 1.6 fold respectively; Fig. 6Bv&vi). Additionally, as we have demonstrated increase MDSC populations in the gp130^757FF^ xIL-1RT1^−/−^ stomach and TNF-α can directly influence MDSC recruitment [28] we measured the expression of Arg-1, S100A8 and S100A9, genes associated with MDSC development and function. In unaffected proximal stomach of gp130^757FF^ xIL-1RT1^−/−^ mice Arg-1, S100A8&9 were decreased compared to wildtype (–3.3 ± 1.0, –3.0 ± 0.6 and –2.3 ± 0.3 fold respectively; Fig. 6Bvii, vii&ix), however all were markedly increased in gp130^FF^ xIL-1RT1^−/−^ cardiac lesions (16.3 ± 6.3, 4.4 ± 2.3 and 5.0 ± 2.8 fold respectively; Fig. 6Bvii, vii&ix). These data suggest that TNF-α expression in the cardiac lesions of gp130^757FF^ xIL-1RT1^−/−^ results in an increase in MDSC recruitment.

To further assessed the effects of TNF-α on the gp130^757FF^ gastric tumor phenotype, gp130^757FF^ mice were crossed with TNF-α^−/−^ mice. Macroscopic assessment of lesion size demonstrated that gp130^757FF^ xTNF-α^−/−^ mice had considerably smaller distal tumors compared to gp130^FF^ mice (38.91 ± 5.46 and 70.16 ± 6.27 mm^2^ respectively; Fig. 6Bi–iii). These data show that TNF-α promotes gp130^757FF^ tumor development, potentially through the recruitment of MDSCs that are able to restrict the anti-tumor response and are thus permissive for tumor growth.

## DISCUSSION

IL-1α and IL-1β mRNA expression is upregulated in antral tumors of gp130^757FF^ mice, consistent with the view that IL-1RT1 ligands may contribute to the development of gastric antral tumors in this STAT3-dependent model, and may serve as novel therapeutic targets for gastric cancer. To test this directly gp130^757FF^ mice were crossed with IL-1RT1^−/−^ mice. Unexpectedly, in the absence of IL-1RT1 signaling there was a significant increase in the size of gp130^757FF^ gastric antral tumors, demonstrating that not only is activation of IL-1 signaling dispensable for tumorigenesis in the distal stomach of gp130^757FF^ mice, but that patent IL-1 signaling pathways can act to restrict tumor growth in the presence of cytokine-driven STAT3-mediated gene transcription.

The negative influence of IL-1RT1 signaling was also observed in the gp130^757FF^ xIL-1RT1^−/−^ proximal stomach where a significant increase in both tumor incidence and size compared to gp130^757FF^ mice was observed. These proximal tumors originated from the gastric cardia at the limiting ridge near the gastro-esophageal and fore-stomach junction. gp130^757FF^ xIL-1RT1^−/−^ cardiac lesions were associated with elevated STAT3 activation, a modulated immune response, including an enrichment of MDSCs, and loss of specialized gastric cells. The clear positive correlation between STAT3 activation and cardiac lesions indicates that STAT3 activation is causative for cardiac tumor development in this model. Furthermore, increased STAT3 activation correlated with increased IL-11 but not IL-6 mRNA expression, suggesting that IL-11 is the main ligand driving gp130 activation in cardiac lesions, as we have previously demonstrated for the primary antral lesions in the gp130^757FF^ mouse model [19]. Additionally, at this advanced stage of pathology the recruitment of immunocytes which express STAT3 inducing genes may also contribute to STAT3 activation. This alternative pathway is supported by our analysis of the mRNA expression of IL-23 and amphiregulin, both of which signal via STAT3 [29, 30] and which were increased in gp130^757FF^ xIL-1RT1^−/−^ cardiac lesions. Nonetheless, IL-11 is absolutely required for gp130^757FF^ gastric tumorigenesis and therefore must be considered the primary initiator of cardiac lesions.

The role of IL-1RT1 signaling during IL-11-induced STAT3 activation without tumor inducing gp130 mutations was also assessed. This was achieved by continuous, systemic, low dose IL-11 administration. IL-1RT1^−/−^ mice treated with IL-11 showed immune and gastric cell marker changes similar to those observed in gp130^757FF^ xIL-1RT1^−/−^ cardiac lesions, whereas wildtype mice treated with IL-11 did not. This demonstrates that IL-11 can promote gastric atrophy, consistent with our previous findings [7] and that IL-1 signaling antagonizes IL-11-dependent tumorigenesis.

Both wildtype and IL-1RT1^−/−^ mice treated with IL-11 had increased STAT3 activation in the proximal stomach and unchanged endogenous IL-11 expression. IL-6 expression on the other hand, was markedly reduced in IL-1RT1^−/−^ mice administered IL-11, suggesting that IL-6 expression is dependent on intact IL-1RT1 signaling. Quante *et al* (2012) [4] suggest that enhanced IL-6-induced STAT3 activation is crucial for the cardiac/esophageal pathology that develops in IL-1β transgenic mice. However they did not measure IL-11 expression, a well-documented inducer of STAT3 phosphorylation whose expression is absolutely required in the gp130^757FF^ mouse [19] and is strongly upregulated in several other models of gastric tumorigenesis [19], including the cardiac lesions in gp130^757FF^ xIL-1RT1^−/−^ mice.

In general we observed the same cardiac pathology and gene expression pattern in mouse genetic models which have either exaggerated IL-1RT1 signaling [4], or ablated IL-1RT1 signaling (gp130^757FF^ xIL-1RT1^−/−^). Therefore it seems that the common thread that links the cardiac pathology in both models is not intact IL-1RT1 signaling *per se*, but augmented expression of IL-6 family cytokines which hyperactivate gp130/STAT3 leading to MDSC recruitment. Together these outcomes highlight the importance of gp130 ligands and dysfunctional IL-1RT1 cytokines in driving upper gastric pathology. Supporting evidence comes from individuals carrying polymorphisms in the IL-1α, IL-1β and IL-1RN gene cluster that lead to elevated expression of IL-1 cytokines, and who are actually protected from gastro-eosphageal reflux disease and Barrett's esophagitis [31–33], suggesting that a delicate balance is at play, with expression of IL-1 cytokines in the proximal stomach and esophagus under some circumstances leading to pathology, and in others being protective. These findings in conjunction with the observed detrimental effects of ablated IL-1 signaling in IL-11/STAT3 mediated gastric pathology demonstrated here, suggest that IL-1 receptor blockade, or immunoneutralisation of ligands may not be suitable targets for preventing gastric cancer progression. Furthermore inhibiting these cytokines in humans may result in more aggressive tumors and possibly enhanced metastasis.

The observation that loss of IL-1RT1 signaling in the gp130^757FF^ mouse produces more pathology was unexpected considering that high expressing IL-1β polymorphic variants correlate with increased gastric cancer risk in Caucasians, and transgenic overexpression of IL-1β in mice results in severe gastric and esophageal pathology [4, 8]. However, while much of the current literature has focused on the pro-tumorigenic effects of IL-1β, particularly in gastric cancer, IL-1α, which was preferentially expressed in gp130^757FF^ xIL-1RT1^−/−^ cardiac lesions at the expense of IL-1β, has been poorly studied in comparison. Although these two cytokines share many functions, IL-1α has a novel role as an alarmin. Alarmins describe a class of multifunctional cytokines released by necrotic cells in response to infection or injury to promote an immune responses [34]. As such, IL-1α is expressed constitutively in the nuclei of cells to be released and activated by cellular damage, thereby initiating an immediate inflammatory response [15, 35, 36]. In mice, the loss of IL-1α, but not IL-1β, results in decreased survival rates in response to lethal endotoxemia [37], and impaired immune function during infection [38–40]. Furthermore, the loss of IL-1 signaling in colitis in mice results in more severe colonic pathology suggesting IL-1 has the capacity to protect against mucosal injury [41, 42]. Many studies identify IL-1α as being a critical component of the early phase recruitment of immunocytes particularly macrophages and neutrophils [43–45]. We suggest that in our model the absence of patent IL-1α alarmin function in the stomach due to IL-1RT1 deletion results in inappropriate activation of cellular infiltrate, ultimately resulting in chronic damage, and enhanced tumorigenesis.

The current dogma supports the view that IL-1β promotes enrichment of MDSCs in the stomach thus balancing the alarmin activity of IL-1α [46], and can lead to gastric tumour formation [8]. Additionally, MDSC numbers are known to be an independent risk factor for disease progression in gastric and esophageal cancer [47, 48]. MDSCs can promote tumor immune tolerance through suppression of T cell responses leading to tumor expansion and disease progression [49, 50]. In the gastrointestinal tract, MDSC-induced tumor immune tolerance has been demonstrated to promote gastric and esophageal cancers [4, 8, 47, 48], suggesting that enrichment of MDSCs directly correlates to gastric and esophageal disease outcomes. Here, we demonstrate that IL-11/STAT3 causes an enrichment of MDSCs independent of IL-RT1 signaling. This finding is consistent with published data from the lung and pancreas [51, 52]. Furthermore, IL-6 signaling, a potent STAT3 inducer, is required for IL-1β-induced gastric and esophageal pathology [4, 8], indicating that STAT3 signaling is necessary for MDSC expansion and explains how both overexpression and ablated IL-1 signaling can induce cardiac pathology through STAT3 dependent MDSC enrichment.

An additional consequence of a deficiency in IL-1RT1 signaling in the gp130^757FF^ stomach is elevated TNFα, also known to promote the enrichment of MDSC populations though expression of S100A8 and S100A9 [52]. One mechanism by which MDSCs may promote tumor growth is through expression of arginase-1, which in turn diminishes T cell mediated anti–tumor responses [48]. Here we show elevated S100A8 and 9 as well as arginase-1, suggesting enhanced MDSC activity in stomach tumors deficient in IL-1RT1 signaling. Moreover the absence of TNFα in gp130^757FF^ mice results in smaller tumors, likely as a result of reduced numbers of MDSCs and an exaggerated pro-inflammatory environment.

Here we have demonstrated that IL-1RT1^−/−^ signaling is not required for IL-11/STAT3 mediated pathology, with genetic depletion of IL-1RT1^−/−^ resulting in a more severe tumor phenotype by promoting an anti-inflammatory, pro-tumorigenic environment. We propose a mechanism involving IL-11-dependent recruitment of tumor promoting MDSCs to the stomach. Furthermore, these data provide additional evidence that gp130-induced STAT3 activation is required for cardiac and esophageal pathology, and may provide the basis for as a future therapeutic target for such pathologies.

## MATERIALS AND METHODS

### Mice

All mice were on a C57Bl/6 background and were used at 12–14 weeks of age. Transgenic mice were genotyped by multiplex PCR as previously described [7, 20]. Ethical approval (#664A) was obtained from the animal ethics committee of the Murdoch Children's Research Institute.

### Cytokine treatment

WT mice and IL-1RT1 mice (*n* > 10) were systemically administered 1μg of recombinant human IL-11 protein (Shenandoah) per day for seven days, or saline, via Mini Osmotic Pumps (MOP) (Alzet Model 1007D) implanted subcutaneously.

### Tissue preparation

Mouse stomachs were prepared and analysed as previously described [21]. Briefly, the stomach was bisected, antrum and fundus from one half were frozen for TRIzol extraction, and the other half fixed in 4% paraformaldehyde in PBS. When obvious proximal (cardiac) stomach lesions were evident, the proximal stomach was further divided into affected and unaffected tissues. Spleens were also collected from MOP mice; one half was frozen for TRIzol extraction and the other fixed in 4% paraformaldehyde in PBS.

### Macrophage isolation and analysis

To harvest peritoneal macrophages, 5 mL of HBSS (Sigma) containing 10 units/mL of heparin (Sigma) was injected into the peritoneal cavity (*n* = 5/group). Extracted cells were harvested, washed, and resuspended in complete RPMI then plated onto 6-well culture plates. Plates were incubated at 37°C for 10 minutes to allow macrophages to adhere. Cultures were then washed to remove non-macrophage cells. Macrophages were incubated overnight at 37°C. The next day, macrophage cultures were treated with either saline or 100 ng LPS/mL in fresh media. Cells were harvest using TRIzol reagent (Life Technologies).

### Quantitative RT-PCR

RNA was harvested using TRIzol reagent (Life Technologies). RNA (3μg) (*n* ≥ 10 animals/group) was reverse transcribed using Moloney murine leukemia virus reverse transcriptase (Promega) primed with oligo (dT). Quantitative RT-PCR (QRTPCR) primers were designed using PRIMER EXPRESS (Applied Biosystems)(Supplementary Table 1). SYBR green chemistry was used with rL32 as the internal reference gene. QRTPCR conditions were 95°C for 10 min, 40 cycles of 95°C for 15 sec and 60°C for 15 sec (Applied Biosystems AB7500). Results were analyzed using sequence detector software, relative fold differences were determined using the ΔΔCt method.

### Immunoblotting

Proteins (*n* ≥ 10 animals/group) were prepared using TRIzol (Life Technologies) and 20μg of extract was subjected to SDS PAGE. Membranes were incubated with specific antibodies (Supplementary Table 2), peroxide-conjugated secondary antibody and visualized by enhanced chemiluminescence (Amersham). Quantification was done using Quantity 1 software (Bio-Rad Laboratories) and ratios of phosphorylated: total protein determined from duplicate membranes.

### IL-1β ELISA assay

TRIzol extracted proteins were diluted to 0.1% SDS using sterile PBS and IL-1β sandwich ELISA was performed as previously described [53, 54].

### Quantitative morphometry

#### Macroscopic

Images were captured on a Coolpix 4500 digital camera (Nikon Instruments, Melville, NY). Quantitative analysis of stomach lesions was done using ImageJ software for Windows v1.38 [55]. Measurements were converted to millimetres after comparison with a calibrated graticule.

#### Microscopic

Images of H&E stomachs were taken along the length of the stomach using a Coolpix 4500 digital camera attached to a light microscope. Lengths and areas were manually traced on these images using ImageJ software (see above). Measurements were converted to millimetres after comparison with a calibrated graticule.

### Cell isolation and flow cytometry (FACS) analysis

#### Stomach cell isolation

Stomachs were collected in HBSS (2mM EDTA, 2% FBS) then perfused with digestion media (1x HBSS [without calcium and magnesium], 5mM EDTA, 5% FBS and 1mM dithiothreitol [DTT]) and incubated at 37°C for 15 minutes. Stomachs were then cut and incubated in digestion media for a further 15 minutes then passed through a 70μm cell strainer. Cell suspensions were stained with fluorescently labeled antibodies (Supplementary Table 3) in HBSS (2mM EDTA, 2% FBS), washed and resuspended in HBSS for FACS analysis (Becton Dicknson LSRII and FACSDiva v6.1.1) or cell sorting (BD Influx cell sorter).

#### Spleen cell isolation

One third of the spleen was made into a single cell suspension. Red blood cells were lysed with Ammonium-Tris Chloride buffer for 5 minutes at room temperature. Cell suspensions were stained with fluorescently labeled antibodies similar to that of the stomach.

#### FACS analysis

Dead, autofluorescent and aggregated cells were gated out of on the basis of FSC, SSC and propidium iodide staining. Live cells were gated based on FSC v SSC plot and the number of events was recorded for the different cell types (Supplementary Table 3). The number of events for each cell type was compared to the total number of all live cell events and proportions of cell types were compared between treatment groups.

### Statistical analysis

All data were expressed as mean ± SEM and statistical analysis was performed by one-way analysis of variance (ANOVA) and the appropriate parametric or nonparametric statistical test using Sigmastat (Jandel Scientific). All *p* values were derived and comparison was performed between the wildtype control group and specific treatment groups unless indicated. *p* values ≤ 0.05 were considered statistically significant.

## SUPPLEMENTARY FIGURES AND TABLES


