# Supplementary material for: IL-1RT1 signaling antagonizes IL-11 induced STAT3 dependent cardiac and antral stomach tumor development through myeloid cell enrichment
Source: Oncotarget. 2015 Jan 6;6(2):679–95. doi: 10.18632/oncotarget.2707 (PMC4359248; doi:10.18632/oncotarget.2707)
Supplement: Supplementary file 1 [file oncotarget-06-679-s001.pdf]

SUPPLEMENTARY FIGURES AND TABLES

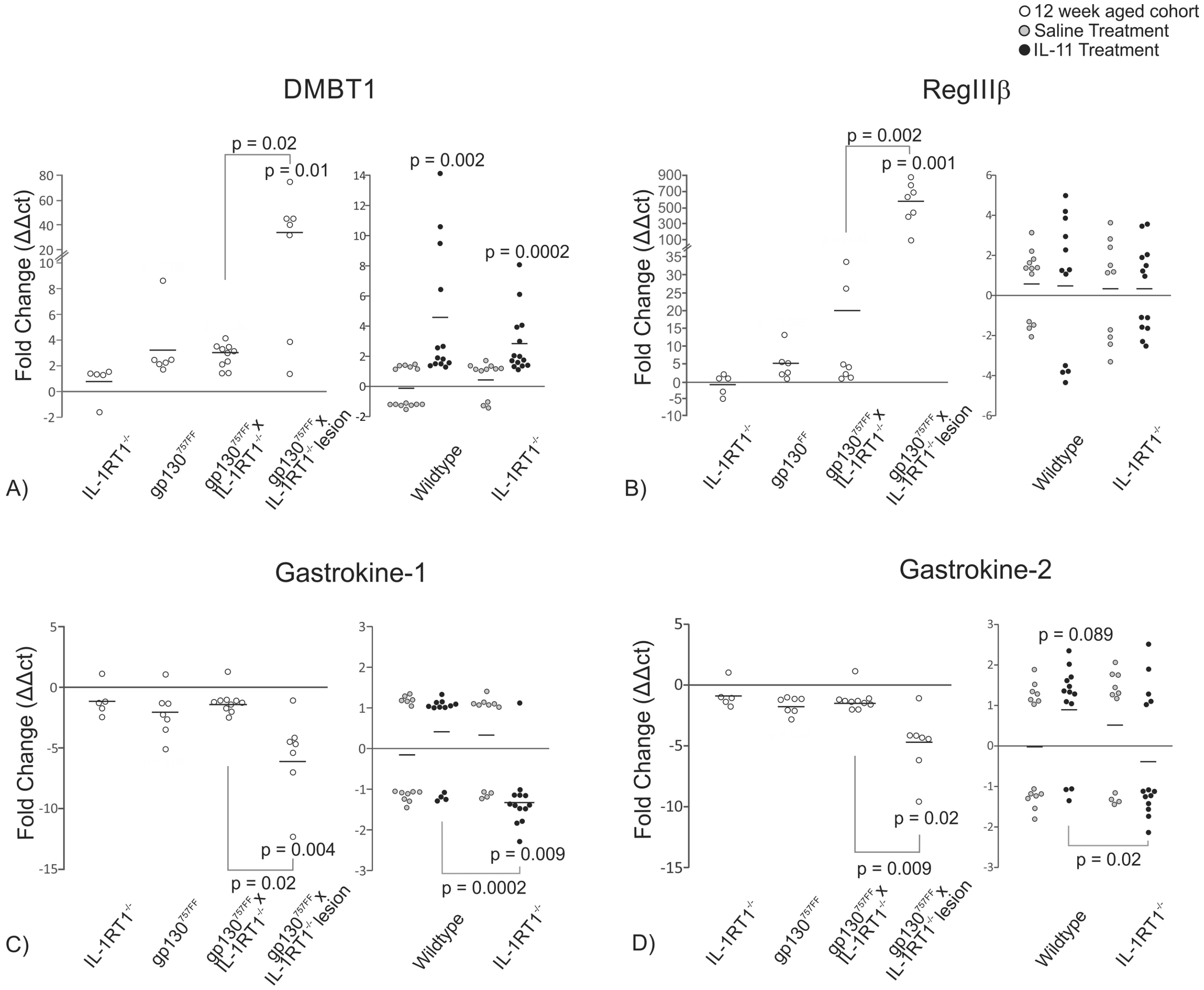

Supplementary Figure 1: Proximal stomach mRNA expression in gp130^{757FF} xIL-1RT1$^{-/-}$ cohort mice and wildtype and IL-1RT1$^{-/-}$ mice constitutively administered 1ug IL-11 daily for 7 days via QRT-PCR. Analysis of gastric inflammation and pathology: **(A)** DMBT1, **(B)** RegIIIβ, **(C)** GKN-1 and **(D)** GKN-2. *p*-values are as indicated for statistically significant changes.

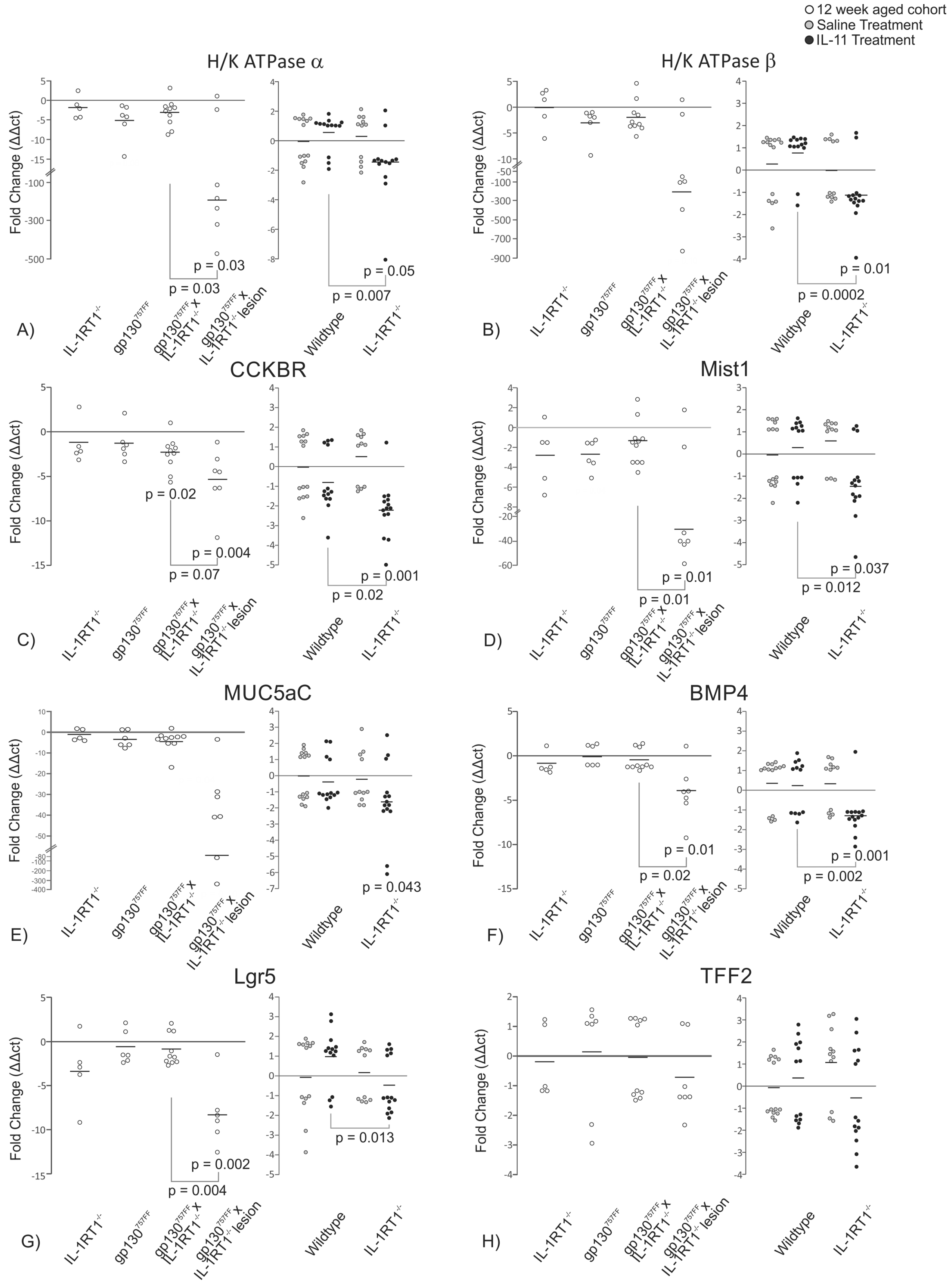

Supplementary Figure 2: Proximal stomach mRNA expression in gp130^{757FF} xIL-1RT1$^{-/-}$ cohort mice and wildtype and IL-1RT1$^{-/-}$ mice constitutively administered 1ug IL-11 daily for 7 days via QRT-PCR. Analysis of gastric cell types using appropriate markers; parietal cells: **(A)** H/K ATPase α, **(B)** H/K ATPase β and **(C)** CCKBR; Chief cells: **(D)** Mist1; Mucus secreting cells **(E)** Muc5aC; Progenitor cells: **(F)** BMP4 and **(G)** Lrg5; and SPEM pathology: **(H)** TFF2. *p*-values are presented for statistically significant changes ($p < 0.05$).

Supplementary Table 1: Primer sequences used for mRNA expression analysis via QRT-PCR in order as presented in results

Gene	Forward Primer	Reverse Primer
Il-1α	AACCCATGATCTGGAAGAGACC	TGGTGCTGAGATAGTGTTTGTCC
IL-1β	CAGGCAGTATCACTCATTGTGG	GTGCAGTTGTCTAATGGGAACG
IL-1RT1	CGGCGCATGTGCAGTTAATA	TGTAGCCGTGAGGATGATAAAGC
IL-1RAP	CCCGATAAGGATGGGACTTCT	AGCGCTCCGAAGCATGACT
IL-1RT2	AAGGTTTCTGAATCCCCCATTAC	TTGAACTCTCTGCCACGAAACTG
IL-1RAN	TCTGACAGTGGAACGGAATGAC	TTGCATCTTGCAGGGTCTTTT
IL-11	CTGCAAGCCCGACTGGAA	AGGCCAGGCGAGACATCA
IL-6	ACAAAGCCAGAGTCCTTCAGAGA	CTGTTAGGAGAGCATTGGAAATTG
DMBT1	GCTCCACAAGCACAGATTCC	TGCCATCTCATTGTTCTTGG
RegIIIβ	GAACCCATCTACTGCCTTAGACC	CTTCACATTTTGTCCCTTGTC
Gastrokine-1	CTTCAGGACCTCGATACAATGG	TTGAGTACAAAGGCTGGTTTGG
Gastrokine-2	AATGTAGACGGAAGTGGACAGC	GCATCCTTGTTCATTCTGTGC
S100A8	GTCCTCAGTTTGTGCAGAATATAAA	GCCAGAAGCTCTGCTACTCC
S100A9	CTCTTTAGCCTTGAAGAGCAAG	TTCTTGCTCAGGGTGTCAGG
MIP2	AGTGAACTGCGCTGTCAATG	TTCAGGGTCAAGGCAAACTT
TNFα	TATGTCTCAGCCTCTTCTCATTCC	ATGATCTGAGTGTGAGGGTCTGG
IL-23	GAGCAACTTCACACCTCCCTACTAGTAG	GGCCTGGAGGCTTCGAA
Amphiregulin	CTATCTTTCTCTGCCATCATC	CAACTTTTACCCTTGCAATTGCC
Arg-1	CTGCATGGGCAACCTGTGT	CCTGGTACATCTGGGAACTTTCC
H/K ATPase α	CAAATATCATCGCCAGCTTCAA	TCCCCGTCTCGGATCACA
H/K ATPase β	CCGGTGGGTGTGGATCAG	GCAAAGAGCCCGGTCATG
CCKBR	ACAAATGTGGTCCGTGCTTCT	GCGGGAGATGAGTCCATAGG
Mist1	CCAGGGTGCTCCTTCTTTTG	GGCGGAAGTTCACCATCCTT
Muc5aC	GCAGTTGTGTCACCATCATCTGTG	GGGGCAGTCTTGACTAACCCTCTT
BMP4	GCCGTCATTCCGGATTACAT	CTCCTCACAGTGTTGGCTCG
Lgr5	GGAATAAAGACGACGGCAACA	GTCCCGCTCATCTTGAACTTG
TFF2	CCCCACAACAGAAAGAAC	GGGCACTTCAAAGATCAG

Supplementary Table 2: Antibodies used for protein detection via immunoblotting

Protein	Antibody	Raised In	Company	Reference No.
β-actin	β-Actin (13E5) Rabbit mAntibody	Rabbit	Cell signaling	#4970
STAT3	STAT3 Antibody	Rabbit	Cell signaling	#9132
p-STAT3	Phospho-STAT3 (Tyr705) Antibody	Rabbit	Cell signaling	#9131
ERK1/2	p44/42 MAP Kinase Antibody	Rabbit	Cell signaling	#9102
pERK1/2	Phospho-p44/42 MAK Kinase Antibody	Rabbit	Cell signaling	#4377
AKT	AKT Antibody	Rabbit	Cell signaling	#9272
pAKT	Phospho-AKT (Ser473) Antibody	Rabbit	Cell signaling	#4058
STAT1	STAT1 Antibody	Rabbit	Cell signaling	#9172
pSTAT1	Phospho-STAT1 (Try-701) Antibody	Rabbit	Cell signaling	#9171
mIL-11	IL-11 Antibody	Mouse	WEHI	3/99-4D9-27-25

Supplementary Table 3: Fluorochromes used for Flow Cytometry analysis

Marker	Fluorochromes	Company	Reference No.
CD4	V450	BD Biosciences	560468
CD8a	APC-Cy7	BD Biosciences	561967
CD11b	PE	BD Biosciences	557397
CD11c	APC	BD Biosciences	550261
CD45R/B220	FITC	BD Biosciences	553087
Ly-6G and Ly6C (Gr-1)	PerCP-Cy5.5	BD Biosciences	552093

Immune Cell	Fluorochromes
MDSC CD11b+Gr-1+	$CD45^{+}/CD11b^{+}/CD11c^{int}/Gr\text{-}1^{int}$
Th CD4+	V450+ / APC-Cy7–
CTL CD8+	V450– / APC-Cy7+
